# A comparison of the scorings of real and standardized patients on physician communication skills

**DOI:** 10.12669/pjms.303.3255

**Published:** 2014

**Authors:** Rita Rezaei, G Mehrabani

**Affiliations:** 1 Rita Rezaei, PhD, Department of Medical Education, Shiraz University of Medical Sciences, Shiraz, Iran.; 2 G Mehrabani, MD, Stem Cell and Transgenic Technology Research Center, Student Research Committee, Shiraz University of Medical Sciences, Shiraz, Iran.

**Keywords:** Patients rating, Physician communication skills

## Abstract

***Objectives:*** To compare the scorings of real and standardized patients on physician communication skills.

***Methods:*** Patient scoring (n=183) on physicians’ communication skills was determined by 93 real and 90 standardized patients. Eighty physicians (42 specialists and 38 general physicians) in private practice were enrolled. Data were analyzed using self administered questionnaires and checklists including 16 close ended questions.

***Results:*** Twelve percent of patients were not satisfied with the physician communication skills. Poor communication skills were more reported by male patients and those with a higher educational level. The physician communication skill received a higher score with increase of age of patients. A good physician’s communication skill was reported more by married patients. A good physician’s communication skill was significantly more in female doctors, in general physicians and in doctors wearing a White Coat. Real patients scored physician’s communication skills higher than standardized patients.

***Conclusion: ***It is important that physicians try to learn the principles of a good physician-patient communication skill. Therefore, providing medical educational programs on the role of a good doctor and patient relationship at all levels for the doctors and applying them in their clinical practice seem necessary to improve the physician communication skills.

## INTRODUCTION

The doctor-patient relationship has always been an important point in health issues and contemporary medical ethics. It is essential in delivery of a high-quality health-care for diagnosis and treatment of diseases. Most of the universities usually start their teaching plans before the entrance of students to the hospitals to provide a professional rapport from the patients considering their dignity and privacy.

The patient care and a good communication skill are the most important dimensions in health-care to ensure their quality.^1^ The patient rating of the physician communication skills is still a predictor for patient satisfaction^2^ and standardized patients are considered as a tool to evaluate the physician communication skills.^3^ Patient care is a core dimension to determine the health care quality while strong communication skills seem necessary to achieve this care^1^ but still most of health policy makers and researchers are not familiar with physician communication skills.^4^ This study was performed to compare the scorings of real and standardized patients on physicians’ communication skills.

## METHODOLOGY

Rating of physician communication skills was undertaken to assess physician communication skills using 93 real and 90 standardized patients (n=183). In a systematic cluster sampling method, 80 physicians (38 general physician and 42 specialists) from private practice in Shiraz, southern Iran were enrolled. 

The standardized patient role was defined to provide physicians with indented clinical scenario and to avoid potential rating biases with real patients. They presented the same signs and symptoms of the disease to the same doctor on the same day. Data were gathered by self administered questionnaires and observation checklists including 16 close ended questions. The validity and reliability of the questionnaires were determined in a pilot study using 150 patients and 30 physicians and data were analyzed by 5 separate doctors using a check list. The questionnaire included data on sex, age, marital status, educational level of patients and doctors, way of salutation, explanations to patients, way of attention to patients and frequency of referrals of patients to doctors. Variables were scored as poor, acceptable and good and a mean of scores was recorded.

In scoring, a good and poor communication skill was defined above and below the mean score respectively. The study was approved by the Ethics Committee of Shiraz University of Medical Sciences. 

SPSS software (Version 11, Chicago, IL, USA) was used for statistical analysis. Chi-square test was applied to compare the findings and a p value less than 0.05 was considered statistically significant.

## RESULTS

From 183 patients who participated in this study, 59 (32.2%) subjects were male and 124 (67.8%) were female (age=35±15 years, range: 12-88 years) while most of them were in 10-30 years old age group (45.8% males and 55.6% females) and 80% of them were married (71.2% of men and 84.7% of women were married) and 20% were single.

Regarding patients’ scoring, 12% were not satisfied with the physician’s communication skills. Poor communication skills were reported more by male patients (*P*>0.05) and those with a higher educational level (42.5%) (p<0.05). A good physician’s communication skill was significantly more in female doctors, in general physicians (p<0.05). A good physician’s communication skill was scored by married patients (64.6%) (p<0.05) (Table 1).

**Table 1:** The correlation between marital status of patients and scoring to a physician’s communication skills

*Scoring*	*Poor * *No. (%)*	*Acceptable* *No. (%)*	*Good* *No. (%)*	*Total* *No. (%)*
Married	12 (8.2)	40 (27.2)	95 (64.6)	147 (100)
Single	10 (27.7)	11 (30.6)	15 (41.7)	124 (100)

According to the patients scoring, 69.4% of female doctors showed a good communication skill. A good communication skill was noticed more among general physicians (66.2%) when compared to specialists (54%) (*P*>0.05). Doctors wearing a white coat were shown to have scored higher communication skills when compared with doctors wearing a casual dress.

According to the standardized patients’ scoring, 47% of doctors received poor communication skills and 50% of female physicians received a good communication skill while this figure among male physicians was 13.8% showing good communication skills among female doctors by standardized patients. There was no significant correlation between specialists and general physicians’ communication skills by these patients. Real patients scored physician communication skills higher (59%) than standardized patients (17%). 

## DISCUSSION

A good physician’s communication skill seems necessary for a doctor to establish a good relationship with the patient. With the increase in demand for doctors, a doctor-patient relationship has become an important area of interest for both medical researchers and administrators affecting both patient satisfaction and health care services.^5^

Scoring of a physician’s communication skill by patients may have some limitations such as selection of the physician by the patient, patient adaptation to the physician's practice by passing time, length of doctor patient relationship, kind of disease and the main complaints.^5^

Our findings showed that real patients scored physician communication skills higher than the standardized patients. This is contrary to the previous reports showing that standardized patients scored physician communication skills more than real patients.^6^

In our study, older patients and patients with lower educational level scored physicians’ communication skills higher which may be due to more knowledge of young and educated people with the patients’ rights. In this study, a significant correlation was noticed between gender of the physicians and patient satisfaction revealing that female doctors had better communication skills in comparison to male doctors that is against Shilling et al.’s findings (2003) revealing no correlation.^7^

Another factor that could affect doctor-patient relationship is wearing of the white dress which was previously described.^7^ Our finding indicated to a significant and positive correlation between patients scoring of physicians’ communication skills and wearing of white dresses. Doctors wearing a white coat scored a higher communication skill when compared with doctors wearing a casual dress. It may be due to wearing of a white dress that could present a professional and academic appearance to confirm an authorization for the job.^8^

The duration of relationship between physicians’ communication skills and patients was demonstrated to affect the patients scoring for the physicians’ communication skills. This study showed that patients with a longer patient and doctor relationship scored the doctors with a higher rate. Patient scoring of physicians’ communication skills can be subjective to a number of limitations including the potential biases of non-responsiveness, patient selection of a physician and patient accommodation to the doctor practice,^9^ length of patient and doctor relationship and patient main complaints.^10^ It was shown that patients responded in a global manner when rating the physicians’ communication skills. The patient scoring of physicians’ communication skills is considered as a key predictor of patient global satisfaction.^11^

Epstein and Hundert (2002) showed that standardized patients represented a potentially more objective means for assessment of physician communication skills.^12^ Standardized patients were demonstrated by some authors to be a reliable trained subjects for scoring of physicians’ communications skills.^13^^,^^14^ Standardized patients can be used to score communication skills such as physicians’ history taking, physical examination, medical decision making, communication, as well as health service utilization and the quality of medical care.^7^ Some researchers have reported that standardized patients can be regarded as a standard reference to assess physician communication skills too.^15^

It is clear from the literature that better physician communication skills can affect the patients’ satisfaction and the clinical outcome of their therapy. As such it is important that physicians try to learn the principles of a good physician-patient communication skill. Therefore, designing medical educational programs on the role of a good doctor and patient relationship at all levels for the doctors and applying them in their clinical practice seem necessary to improve the physician communication skills.
